# MicroRNA Biogenesis Pathway Gene Variants Are Associated with Prostate Cancer Susceptibility

**DOI:** 10.3390/ijms27125578

**Published:** 2026-06-20

**Authors:** Irina Gilyazova, Yanina Timasheva, Elizaveta Ivanova, Galiya Gimalova, Adel Izmailov, Gulshat Abdeeva, Murat Dzaubermezov, Zhanna Balkhiyarova, Inga Prokopenko, Valentin Pavlov, Elza Khusnutdinova

**Affiliations:** 1 Institute of Biochemistry and Genetics, Ufa Federal Research Centre of the Russian Academy of Sciences, 450054 Ufa, Russiaekhusnutdinova@bashgmu.ru (E.K.); 2 Institute of Urology and Clinical Oncology, Department of Medical Genetics and Fundamental Medicine, Bashkir State Medical University, 450008 Ufa, Russia; gimalov@anrb.ru (G.G.); gulshatik2001@mail.ru (G.A.); pavlov@bashgmu.ru (V.P.); 3 Laboratory of Population and Medical Genetics, Ufa University of Science and Technology, 450076 Ufa, Russia; 4 Department of Clinical & Experimental Medicine, University of Surrey, Guildford GU2 7XH, UK; z.balkhiiarova@surrey.ac.uk (Z.B.); i.prokopenko@surrey.ac.uk (I.P.)

**Keywords:** prostate cancer, genetic risk score, miRNA

## Abstract

Prostate cancer (PrC) is one of the most common malignancies among men worldwide. However, the contribution of genetic variation in microRNA (miRNA) biogenesis pathway genes to PrC susceptibility remains poorly characterized in many ethnically diverse populations. We conducted a case–control study involving 532 PrC patients and 550 controls from the Volga-Ural region of Eurasia to evaluate the association of twenty-one single nucleotide polymorphisms (SNPs) with PrC risk using single-variant and polygenic approaches. Association analyses identified rs595055 in the *AGO1* gene as significantly associated with PrC risk after correction for multiple testing. To evaluate the cumulative effect of genetic variation, weighted and unweighted polygenic risk scores (PRSs) were constructed. The weighted PRS was significantly associated with PrC risk (odds ratio per standard deviation increase = 1.63, 95% CI [1.43–1.85], *P* = 1.37 × 10^−13^), and demonstrated moderate discriminatory performance (AUC = 63.1%), outperforming the unweighted model. Individuals in the highest PRS quartile had approximately threefold higher odds of PrC than those in the lowest quartile. Combining the weighted PRS with prostate-specific antigen improved discrimination (AUC = 68.1%). These findings support the contribution of miRNA biogenesis pathway genes to PrC susceptibility and highlight the potential value of pathway-based polygenic risk stratification in understudied populations.

## 1. Introduction

**Figure 1:**
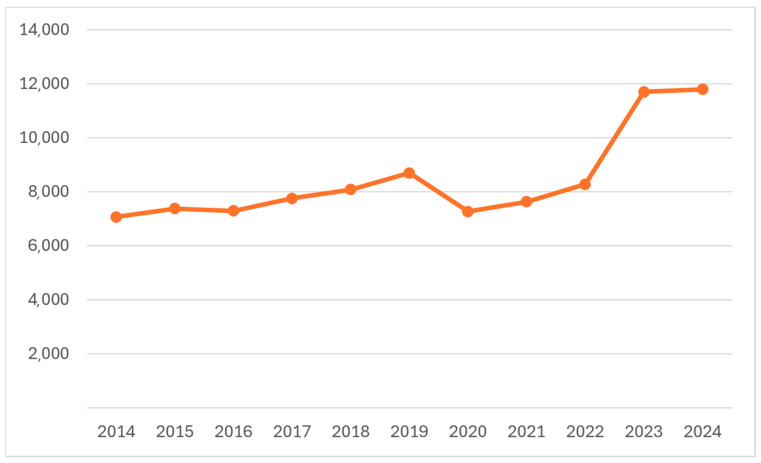
Prostate cancer incidence in the Volga-Ural region of Russia in 2014–2024.

Prostate cancer (PrC) is among the most common malignancies in middle-aged men worldwide. In 2022 alone, approximately 1,467,854 new cases were diagnosed globally, leading to an estimated 397,430 deaths and underscoring the substantial public health burden of the disease [1]. This upward trend is mirrored at the national and regional levels. In the Volga-Ural region, the incidence of PrC rose steadily from 2014 to 2020 and again from 2021 to 2024 (Figure 1), increasing from 7060 to 11,792 annual cases. This corresponds to a 67% overall rise and an average annual growth rate of 5.3% [2]. The lower number of PrC cases recorded in 2021–2022 is likely attributable to the disruption caused by the COVID-19 pandemic [3].

Current approaches to PrC risk assessment rely heavily on prostate-specific antigen (PSA) testing and clinical evaluation. Although PSA remains an important biomarker, its specificity is limited, and additional tools capable of improving risk stratification are needed.

Small non-coding RNAs play a critical role in oncogenesis, and microRNAs (miRNAs) are among the most extensively studied members of this class. These molecules regulate gene expression post-transcriptionally by targeting messenger RNAs for degradation or translational repression. Genomic studies, including genome-wide association studies (GWAS) and the 1000 Genomes Project, have identified approximately 10 million polymorphisms, around 90% of which reside in non-coding regions, including sequences encoding non-coding RNAs [4]. Despite these advances, predicting the functional impact of such genome-wide associations remains challenging. The precise biogenesis of miRNAs is essential for maintaining cellular homeostasis, and disruption of this process is strongly implicated in the development of PrC. This multi-step pathway involves several key genes, including *DROSHA*, *DGCR8*, *XPO5*, *DICER1*, and *AGO2* [5].

Accumulating evidence indicates that specific SNPs in these genes can modulate cancer risk. Meta-analyses have revealed significant associations: the rs417309 variant in *DGCR8* and the rs11077 variant in *XPO5* are linked to an elevated overall cancer risk [6,7], while the rs10719 variant in *DROSHA* is associated with increased risk in Asian populations and has been functionally validated to disrupt miRNA binding and upregulate *DROSHA* expression [8]. Furthermore, although polymorphisms rs3742330 in *DICER1* and rs4961280 in *AGO2* may not affect PrC susceptibility, they are significantly associated with disease progression and aggressiveness [9,10]. For instance, the heterozygous GC genotype of rs2740348 in *GEMIN4* is linked to a reduced risk of developing PrC [11], whereas the CC genotype of rs3746444 in *MIR499* was associated with an elevated risk in an Iranian population [12]. Nevertheless, since the effect of most susceptibility variants is small, increasing attention has been directed toward polygenic risk scores (PRSs), which aggregate the cumulative contribution of multiple variants into a single measure of genetic susceptibility. An alternative to genome-wide PRSs is the construction of pathway-based scores that focus on biologically related genes. Such models may provide mechanistic insight into disease pathogenesis while retaining predictive utility.

Population-specific variation in PrC susceptibility genes points to a substantial ethnic component in the molecular basis of the disease. Thus, identifying polymorphic variants that contribute to PrC development and harnessing modern statistical methods are important priorities for cancer research across diverse populations.

The Volga-Ural region has been a crossroads of human migration for centuries, a history reflected in the genetic structure of its major populations: Russians, Bashkirs, and Tatars, each characterized by a distinct admixture of European and Asian ancestry components. The predominant genetic component derives from Western and Eastern European lineages, with substantial contributions from East Asian (particularly Siberian and Central Asian) populations [13]. These three ethnic groups belong to different language families: Russians are a Slavic group of the Indo-European family, whereas Bashkirs and Tatars belong to the Kipchak sub-branch of the Turkic family. Despite the genetic diversity of the Volga-Ural region, data on genetic susceptibility to PrC in these populations remain limited. In particular, the contribution of variation in miRNA pathway gene factors to PrC risk has not been systematically investigated in the region.

Based on all the above considerations, we hypothesized that polymorphisms in miRNA biogenesis pathway genes collectively contribute to PrC susceptibility. To test this hypothesis, we investigated the association of 30 SNPs with PrC risk in the ethnically diverse populations of the Volga-Ural region and evaluated their cumulative effects using pathway-based polygenic risk score (PRS) models. In addition, we assessed the discriminatory performance, risk-stratification capacity, and robustness of these models through internal validation and comparison with prostate-specific antigen (PSA)-based prediction.

## 2. Results

### 2.1. Study Population and Quality Control

The study initially included 1082 individuals from the Volga-Ural region of Eurasia. Following quality control procedures, eight individuals were excluded because of excessive genotype missingness (>20%), resulting in the final dataset of 1074 participants, comprising 528 PrC patients and 546 controls. Of the 30 genotyped SNPs, 27 passed SNP-level quality control and were retained for subsequent analysis.

The mean age was comparable between cases and controls (Table 1). Among patients with PrC, 55.7% (*n* = 296) had localized disease (TNM stage I–II) and 44.3% (*n* = 236) had advanced disease (TNM stage III–IV). According to Gleason score, 59.6% (*n* = 317) of tumors were classified as Gleason < 8, and 40.4% (*n* = 215) as Gleason ≥ 8. Prostate-specific antigen (PSA) measurements were available for all patients, and for a subset of controls.

### 2.2. Single-Variant Association Analysis

Association results for all 27 SNPs under the unadjusted, age-adjusted, ethnicity-adjusted, and age plus ethnicity-adjusted logistic regression models are provided in Supplementary Table S1. As age and ethnicity were considered the principal confounding factors in our population, subsequent analyses were based on the age- and ethnicity-adjusted model, the results of which are summarized in Table 2.

Among the 27 SNPs that passed quality control, rs595055 in *AGO1* was significantly associated with PrC susceptibility after adjustment for age and ethnicity and correction for multiple testing (OR = 0.64, 95% CI 0.52–0.79, P_FDR_ < 6.72 × 10^−4^). Several additional variants, including rs11060845 (*PIWIL1*), rs7813, and rs2740348 (*GEMIN4*), showed evidence of nominal association (*p* < 0.05), but did not remain significant after FDR adjustment.

### 2.3. Results of the Polygenic Risk Score Analysis

#### 2.3.1. PRS Association with PrC Risk

**Figure 2:**
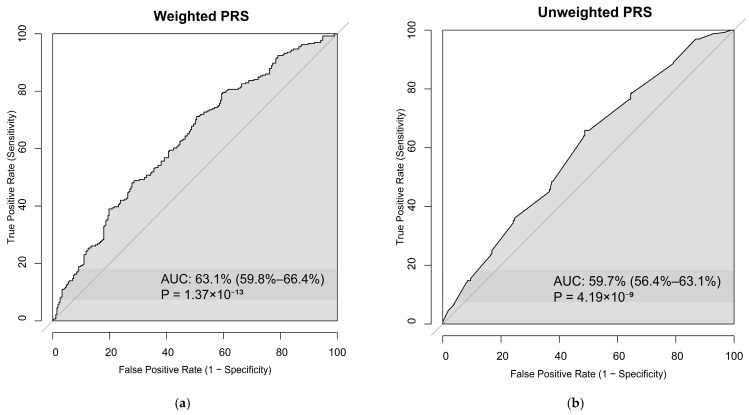
Receiver operating characteristic (ROC) analysis of weighted and unweighted polygenic risk score (PRS) models for prostate cancer. (**a**) Weighted PRS model (AUC = 63.1%). (**b**) Unweighted PRS model (AUC = 59.7%). AUC, area under the curve; P, level of significance.

A weighted PRS and an unweighted PRS were constructed using SNP effect estimates obtained from the age- and ethnicity-adjusted association model. A weighted PRS constructed from QC-passing SNPs was significantly associated with PrC risk (OR per SD increase = 1.63, 95% CI 1.43–1.85, P = 1.69 × 10^−13^). The corresponding unweighted PRS showed a weaker association (OR = 1.46, 95% CI 1.29–1.66, P = 4.05 × 10^−9^). The weighted PRS demonstrated moderate discriminatory ability (AUC = 63.1%, 95% CI [59.8–66.4%]) (Figure 2a), whereas the unweighted PRS showed lower performance (AUC = 59.7%, 95% CI [56.4–63.1%]) (Figure 2b).

#### 2.3.2. Risk Stratification Across PRS Quartiles

To evaluate the relationship between cumulative genetic burden and PrC risk, individuals were stratified into quartiles according to their weighted and unweighted PRS. The lowest PRS quartile (Q1) was used as the reference category, and odds ratios were estimated for each successive quartile (Supplementary Table S2). In addition, a trend analysis was performed to assess whether PrC risk increased progressively across the PRS distribution.

**Figure 3:**
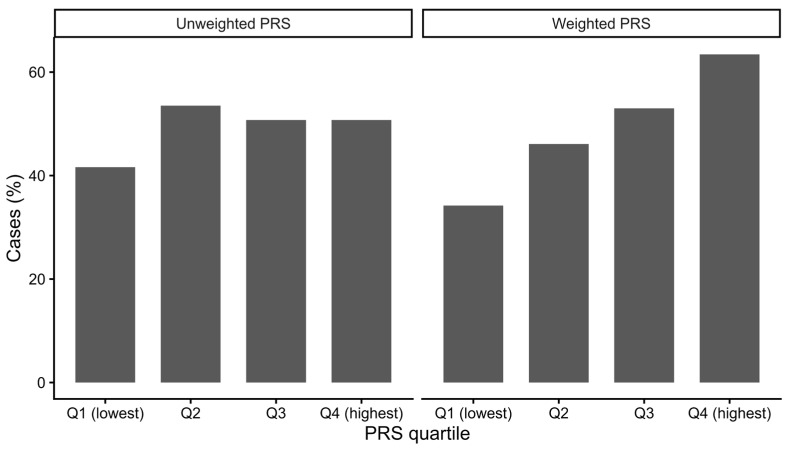
Proportion of prostate cancer cases across quartiles of weighted and unweighted polygenic risk scores (PRSs). Individuals were stratified into quartiles according to their PRS values, from the lowest (Q1) to the highest (Q4) genetic risk category.

A clear dose–response relationship was observed for the weighted PRS (Figure 3). Compared with individuals in the lowest quartile, those in the highest quartile had approximately threefold higher odds of PrC. The odds of PrC increased significantly with each successive quartile (OR per quartile increase = 1.44, 95%CI [1.28–1.63], Ptrend = 2.9 × 10^−9^), indicating a progressive increase in disease risk with increasing genetic burden. The corresponding association was weaker for the unweighted PRS, supporting the use of effect-size weighting when combining variants into a polygenic risk score.

#### 2.3.3. Internal Validation Using Repeated Stratified Training-Validation Splits

To assess the robustness of the PRS models and reduce the risk of overfitting, the dataset was repeatedly partitioned into training (70%) and validation (30%) subsets using stratified random sampling based on disease status and ethnicity. SNP effect estimates were derived from each training dataset and subsequently applied to the corresponding validation dataset.

Across ten independent training-validation iterations, the weighted PRS consistently demonstrated better discriminatory performance than the unweighted PRS (Table 3). In the validation datasets, the weighted PRS achieved a median AUC of 0.553 compared with 0.547 for the unweighted PRS (Supplementary Table S3). Incorporation of the PRS into models containing age and ethnicity resulted in a modest improvement in discrimination, increasing the median validation AUC from 0.660 for the age- and ethnicity-adjusted model alone to 0.669 for the weighted PRS model and 0.670 for the unweighted PRS model.

The relatively small difference between training and validation performance across repeated iterations indicates that the predictive performance of the PRS models was not driven by a single random partition of the dataset, and supports the stability of the observed associations.

#### 2.3.4. The Results of Sensitivity Analysis Using Externally Derived GWAS Effect Estimates

To assess the influence of in-sample weight estimation, an additional PRS was constructed using externally derived effect estimates from published PrC GWAS summary statistics (Supplementary Table S4). External weights were available for 18 of the 27 variants that passed quality control, including one proxy variant.

The GWAS-weighted PRS was associated with PrC status in the unadjusted analysis (OR per SD increase = 0.88, 95% CI [0.78–0.99], P = 0.032), although the association was attenuated after adjustment for age and ethnicity. The discriminatory performance of the GWAS-weighted PRS alone was modest (AUC = 53.9%). Inclusion of the GWAS-weighted PRS in a model containing age and ethnicity increased the AUC from 66.0% to 67.1%.

Overall, the GWAS-weighted PRS showed weaker discrimination than the internally weighted PRS, which is not unexpected given the limited overlap between the candidate variants investigated in the present study and established PrC GWAS loci, as well as potential differences in genetic architecture between the predominantly European GWAS populations and the ethnically diverse Volga-Ural cohort.

#### 2.3.5. Comparison with Prostate-Specific Antigen

**Figure 4:**
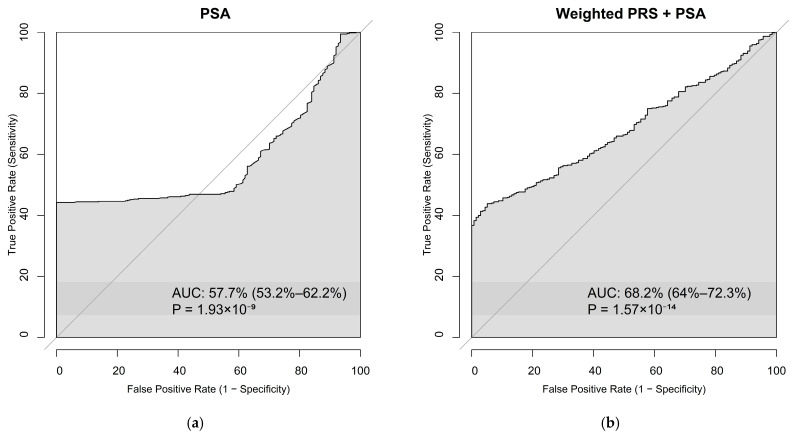
Receiver operating characteristic (ROC) analysis of PSA and weighted polygenic risk score (PRS) models in the subset of participants with available PSA measurements. (**a**) PSA alone. (**b**) Combined PSA + weighted PRS model. AUC, area under the curve; PSA—prostate-specific antigen; P—level of significance.

To compare the discriminative ability of the PRS model and prostate-specific antigen (PSA), we evaluated the diagnostic value of the standard PSA test in our cohort. PSA measurements were available for all cases but only a subset of controls (n = 137). Therefore, PSA analyses were performed in this restricted subset. PSA alone yielded an AUC of 57.7% (95% CI [53.2–62.2%]) (Figure 4a). The weighted PRS demonstrated comparable performance (AUC = 60.7%, 95% CI [55.4–66.1%]). The model incorporating both PSA and the weighted PRS yielded an AUC of 68.2% (95% CI [64.0–72.3%]) (Figure 4b).

### 2.4. Pathway Enrichment Analysis Results

**Figure 5:**
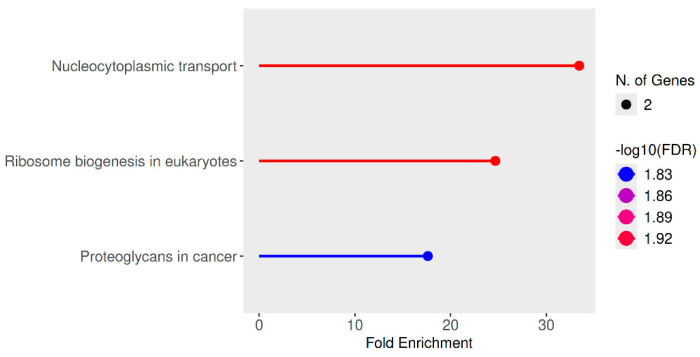
Pathway enrichment analysis of genes harboring prostate cancer-associated SNPs. The bar graph depicts the fold enrichment for the three most significantly overrepresented pathways. SNPs in genes encoding key molecules of ribosome biogenesis and nucleocytoplasmic transport can disrupt the modification of rRNA and lead to activation of oncogenic cell survival pathways [14]. Proteoglycans modulate the expression and activity of cytokines, chemokines, growth factors, and adhesion molecules, and thus influence the behavior of cancer cells and their microenvironment during the progression of tumors. Consequently, alterations in genes related to these pathways can affect tumor growth and progression [15].

To elucidate the biological context of the identified PrC-associated SNPs, we performed a pathway enrichment analysis using the ShinyGo database. This analysis revealed a significant overrepresentation of our gene set in three key pathways (Figure 5). The most enriched was *Nucleocytoplasmic Transport* (highest fold enrichment), followed by *Ribosome Biogenesis in Eukaryotes* (comparable fold enrichment) and *Proteoglycans in Cancer*.

## 3. Discussion

In this study, we evaluated the contribution of genetic variation in miRNA biogenesis pathway genes to PrC susceptibility in the understudied population of the Volga-Ural region using both single-variant and polygenic approaches. Although only one SNP, rs595055 in *AGO1*, remained significantly associated with PrC risk after correction for multiple testing, the weighted PRS demonstrated a statistically significant association with the disease and moderate discriminatory performance. Individuals in the highest PRS quartile had approximately threefold higher odds of PrC than those in the lowest quartile, indicating a clear gradient of genetic risk across the population. Furthermore, the weighted PRS consistently outperformed the unweighted score across repeated stratified training-validation analyses, supporting the contribution of cumulative genetic variation within the miRNA biogenesis pathway to PrC susceptibility.

Comparison with PSA showed that the weighted PRS and PSA exhibited broadly similar discriminatory performance when evaluated in the subset of participants with available PSA measurements. The model incorporating both predictors achieved higher discrimination than either PSA or PRS alone, suggesting that genetic variation within miRNA biogenesis pathway genes captures information not fully reflected by PSA measurements. Although the discriminatory performance of the combined model remains insufficient for clinical implementation as a stand-alone diagnostic tool, these findings support further evaluation of pathway-based PRSs in larger cohorts and in combination with established clinical risk factors, additional molecular biomarkers, and established PrC susceptibility loci identified through large-scale GWAS, as well as with contemporary diagnostic approaches such as multiparametric MRI and PSMA-PET imaging [16].

Recent studies have highlighted the utility of pathway-based PRSs for investigating the biological mechanisms underlying complex diseases and identifying gene networks contributing to disease susceptibility [17]. Although GWAS have identified more than 260 PrC susceptibility loci, most individual variants confer only modest effects on disease risk [18]. Consequently, aggregating the cumulative effects of multiple variants has become a widely used strategy for improving risk stratification. This is illustrated by large GWAS-based PRSs, in which men in the highest PRS categories exhibit substantially elevated PrC risk compared with the average-risk population [19]. The approximately threefold increase in risk observed between the highest and lowest PRS quartiles in the present study is consistent with this broader pattern of genetic risk stratification, despite the substantially smaller number of variants included in our pathway-based model. Unlike conventional GWAS-derived PRSs, the present study focused on genetic variation within the miRNA biogenesis pathway, which was selected on the basis of its established role in post-transcriptional gene regulation and prostate carcinogenesis. The observed association between the weighted PRS and PrC risk supports the hypothesis that coordinated disruption of functionally related genes may contribute to disease susceptibility even when individual variants exert only modest effects.

The clinical utility of PRSs has been demonstrated most clearly in studies integrating common genetic variants with established high-risk susceptibility genes such as *BRCA1* and *BRCA2.* Previous investigations have shown that PRSs can further refine risk estimates among *BRCA* mutation carriers and improve the identification of individuals at increased risk of aggressive disease [20,21,22]. Together with evidence from large GWAS-based studies, these findings support the broader potential of polygenic approaches for personalized risk assessment. Although the present pathway-based PRS incorporated substantially fewer variants than contemporary genome-wide models, it demonstrated significant risk stratification within the study population, supporting continued efforts to develop biologically informed and population-specific PRSs for PrC.

The moderate discriminatory performance observed in the present study is consistent with reports from other PrC PRS investigations. Larger GWAS-based PRSs incorporating hundreds of susceptibility variants generally achieve stronger discrimination than candidate gene models owing to their broader genomic coverage [23,24]. In our study, a sensitivity analysis using externally derived GWAS effect estimates yielded weaker performance than the internally weighted PRS. This result is not unexpected given the limited overlap between the investigated miRNA pathway variants and established GWAS loci, as well as potential differences in genetic architecture between predominantly European GWAS populations and the ethnically diverse Volga–Ural cohort. Nevertheless, the GWAS-weighted analysis provided an important external benchmark and demonstrated that cumulative genetic variation within the miRNA biogenesis pathway contributes to PrC risk beyond individual SNP associations.

Since the contribution of most individual variants to PrC risk is modest, we hypothesized that their cumulative effect might be mediated through disruption of coordinated molecular pathways. The pathway enrichment analysis supports this interpretation, identifying significant enrichment of genes involved in nucleocytoplasmic transport, ribosome biogenesis, and proteoglycan-related pathways. Given the growing interest in miRNA-based therapeutic strategies and computational approaches for identifying miRNA-related molecular interactions, further investigation of these pathways may also provide insights into potential targets for precision medicine approaches in PrC [25,26,27]. These processes have previously been implicated in PrC development and progression and provide a biologically plausible framework linking variation in miRNA biogenesis pathway genes to disease susceptibility [28]. The observation that a pathway-based PRS remained associated with PrC risk despite the limited number of individually significant variants further supports the value of analyzing functionally related gene networks rather than isolated loci.

### Study Limitations

Several limitations should be considered when interpreting these findings. First, the study was conducted in a single geographical region and included a moderate sample size. Second, miRNA expression data were not available, preventing assessment of the functional consequences of the identified variants or direct integration of genetic and transcriptomic information. Third, information on pathogenic variants in established prostate cancer susceptibility genes, such as *BRCA1*, *BRCA2*, and other DNA damage repair genes, was unavailable. Finally, functional validation experiments were beyond the scope of the present study. Future investigations integrating genomic, transcriptomic, and experimental approaches will be important for clarifying the biological mechanisms underlying the observed associations.

## 4. Materials and Methods

### 4.1. Study Sample

The study cohort comprised 532 PrC patients and 550 control individuals from the Volga-Ural region of Eurasia. All PrC patients had histologically confirmed PrC and underwent radical prostatectomy at the Bashkir State Medical University Clinic. Patients were treatment-naïve at enrolment. Consecutive eligible patients were recruited without restrictions based on age, ethnicity, tumor stage, Gleason score, or other clinicopathological characteristics. Control individuals had no history of malignant disease at enrolment. Pre-treatment serum PSA measurements were available for all PrC cases; PSA measurements from routine health check-ups were available for a subset of control individuals. The study was conducted in accordance with the ethical principles of the Declaration of Helsinki [29] and was approved by the Research Ethics Committee of the Institute of Biochemistry and Genetics, Ufa Federal Research Center, Russian Academy of Sciences (approval code #11, dated 27 October 2014). Written informed consent was obtained from all participants.

### 4.2. Sample Preparation, SNP Selection and Genotyping

Peripheral blood samples were collected in 7 mL vacutainer tubes from PrC patients and control individuals. DNA extraction and genotyping were performed as described previously [30]. The selection of polymorphic variants for this study was based on an integrative approach combining biological relevance, bioinformatic annotation, and evidence from previously published studies. Genes involved in miRNA biogenesis and processing pathways were identified through a review of current literature on miRNA regulation and PrC pathogenesis. Candidate variants were selected from genes encoding key components of the miRNA machinery, including *DROSHA*, *DGCR8*, *XPO5*, *RAN*, *DICER1*, *GEMIN3*, *GEMIN4*, *AGO1*, *AGO2*, and *PIWIL1*, as well as from precursor miRNA sequences with potential functional relevance.

Information on SNP location, allele frequencies, and genomic annotation was obtained from the International HapMap Project, dbSNP, miRBase, and Ensembl databases [31,32,33,34]. Variants were prioritized if they were located within coding regions, untranslated regions, regulatory regions, or precursor miRNA sequences where they could potentially influence miRNA biogenesis, maturation, target recognition, or gene expression. Preference was given to polymorphisms with reported or predicted functional effects, including variants affecting miRNA processing efficiency, miRNA–miRNA interactions, or expression of genes involved in miRNA regulatory pathways.

In addition, a systematic review of published association studies was performed to identify variants previously implicated in PrC susceptibility or other malignancies. Variants with minor allele frequencies sufficient for genetic association analysis in Eurasian populations and with available genotyping assays were prioritized for inclusion. Based on this integrative evaluation, a panel of SNPs in miRNA biogenesis pathway genes and precursor miRNAs was selected for genotyping and subsequent association analysis.

### 4.3. Quality Control

Quality control was performed at the sample and SNP levels. Disease status information was available for all participants; therefore, no individuals were excluded because of missing phenotype data. Sample-level missingness was assessed across the 30 genotyped loci, and individuals with >20% missing genotypes were excluded (this criterion corresponds to missing genotype calls across more than six loci). Duplicate identifiers and implausible clinical values (age, PSA concentration, Gleason score, and TNM stage) were assessed during quality control; no such inconsistencies were identified. To evaluate genotyping accuracy, 5% of samples were randomly selected for repeat genotyping, and complete concordance was observed between the original and repeated genotype calls.

SNP-level quality control included the assessment of genotype call rate and Hardy–Weinberg equilibrium (HWE) in controls. SNPs with a call rate < 95% or a HWE P < 0.001 in controls were excluded. HWE testing and genotype quality control procedures were performed using PLINK v1.9 [35]. Of the 30 genotyped SNPs, 27 passed SNP-level quality control and were retained for association analysis.

### 4.4. Power Analysis

Statistical power was assessed prior to association analyses using the Genetic Power Calculator [36] and Quanto v1.2.4 [37]. Calculations assumed a case–control design, PrC prevalence of 8%, and a two-sided significance threshold of α = 0.05. The Genetic Power Calculator was used to estimate power across a range of risk allele frequencies (0.1–0.5) and odds ratios (OR = 1.1–1.5), representing effect sizes commonly reported in gene association studies. The resulting power estimates are presented in Table 4.

Additional calculations were performed in Quanto under dominant, recessive, and log-additive inheritance models (Supplementary text file S1). Assuming approximately 500 individuals per study group, the study provided ≥ 80% power to detect OR ≥ 1.5 for alleles with frequencies ≥ 0.15. Under dominant and log-additive models, ≥80% power was achieved for OR ≥ 1.4 at allele frequencies of at least 0.25 and 0.15, respectively. Under a recessive model, ≥80% power was achieved for OR ≥ 1.7 at allele frequencies ≥ 0.35. Consequently, the study was adequately powered to detect moderate genetic effects but had limited power to detect small effect sizes (OR <1.3).

### 4.5. Statistical Analysis of Individual Genetic Variants

Association analyses were performed under an additive genetic model using logistic regression implemented in PLINK 1.9 [35]. Unadjusted, age-adjusted, ethnicity-adjusted, and age plus ethnicity-adjusted models were evaluated. Ethnicity was grouped into four categories (Bashkir, Russian, Tatar, and Other) and incorporated into regression models as a categorical covariate using indicator variables. Adjustment for multiple testing was performed using the Benjamini–Hochberg false discovery rate (FDR) [38], and adjusted P_FDR_ values < 0.05 were considered statistically significant.

### 4.6. Polygenic Risk Score Analysis

Polygenic risk scores (PRSs) were constructed using the variants that passed quality control procedures. The weighted PRS was calculated using SNP weights derived from the natural logarithm of the OR from the age plus ethnicity association model (lnOR). The unweighted PRS was calculated by assigning each allele a weight of one. For variants with OR < 1, the alternative allele was designated as the risk allele to ensure consistent orientation of SNP effects across loci.

PRS values were calculated using PLINK 1.9 and standardized to z-scores before statistical analyses. Associations between PRS and PrC status were evaluated using logistic regression and reported as ORs per one standard deviation increase in PRS, with 95% confidence intervals and corresponding *p* values. Risk stratification was assessed by grouping individuals into quartiles according to their PRS values. ORs were estimated across quartiles using the lowest quartile as the reference category, and trend tests were performed to evaluate changes in PrC risk across the PRS distribution.

Internal validation was performed using the dataset randomly divided into training (70%) and validation (30%) subsets by stratified sampling based on disease status and ethnicity. SNP effect estimates were obtained from age- and ethnicity-adjusted logistic regression models fitted in the training subset. The resulting effect estimates were used to derive SNP weights for PRS construction, which were subsequently evaluated in the validation subset. To assess the robustness of the findings, the entire training-validation procedure was repeated across ten independent 70/30 stratified splits of the dataset, and summary performance metrics were calculated across validation iterations.

### 4.7. Sensitivity Analysis Using Externally Derived GWAS Effect Estimates

A sensitivity analysis was performed using externally derived effect estimates from published PrC GWAS summary statistics. GWAS information was available for 20 SNPs from the original genotyped panel. One additional GWAS variant, rs11652959, was used as a proxy for the genotyped SNP rs3744741 because of strong linkage disequilibrium between the two variants (r^2^ = 0.8517). Two SNPs with available GWAS effect estimates, rs563002 and rs2292832, were excluded because they failed SNP-level quality control in the present dataset. Therefore, the final GWAS-weighted PRS was based on 18 variants that passed QC. GWAS effect alleles were harmonized to the allele coding in the study dataset before PRS calculation. Published GWAS beta coefficients were used as SNP weights. Associations between the GWAS-weighted PRS and PrC status were evaluated using logistic regression and reported as ORs per one standard deviation increase in PRS.

### 4.8. Receiver Operating Characteristic Analysis

Discriminatory performance was evaluated using receiver operating characteristic (ROC) analysis implemented in the pROC package for R [39]. Predicted probabilities from logistic regression models were used to construct ROC curves. Areas under the ROC curve (AUCs) and corresponding 95% CIs were calculated for PRS-only models and for models including age and ethnicity. For the repeated training-validation analysis, AUCs were calculated separately for each iteration and then summarized across the ten validation splits. Serum PSA measurements were available for all PrC cases and for 137 control individuals; therefore, analyses involving PSA were restricted to participants with available PSA data. PSA-only, PRS-only, and combined PSA + PRS models were evaluated. Differences between AUCs were evaluated using DeLong’s test implemented in the pROC package.

### 4.9. Pathway Enrichment Analysis

To functionally annotate the list of PrC-associated genes identified in our analysis, we performed a comprehensive enrichment analysis using a multi-step bioinformatics approach. The list of candidate genes was submitted to ShinyGO v.0.80, a graphical gene set enrichment tool, to obtain a broad overview of significantly overrepresented biological pathways. The analysis was performed with the following parameters: a false discovery rate (FDR) cutoff of <0.05 and a minimum pathway size of two genes.

This integrated pipeline allowed us to transition from a simple list of candidate genes to a functionally annotated network, highlighting the key biological processes and molecular interactions potentially disrupted in PrC pathogenesis.

## 5. Conclusions

In this study, we demonstrated that genetic variation within miRNA biogenesis pathway genes contributes to PrC susceptibility in the Volga-Ural population. Although only one individual variant remained significantly associated with PrC risk after correction for multiple testing, a pathway-based weighted PRS was significantly associated with disease status and provided moderate discriminatory performance. The weighted PRS consistently outperformed the unweighted model, showed a clear dose-dependent increase in PrC risk across PRS quartiles, and remained robust across repeated training-validation analyses.

The addition of prostate-specific antigen (PSA) measurements further improved risk discrimination, suggesting that genetic and clinical information may provide complementary insights into PrC risk. Sensitivity analyses using externally derived GWAS effect estimates supported the relevance of cumulative genetic variation within the miRNA biogenesis pathway, while also highlighting the importance of population-specific genetic architecture. Several limitations including the moderate sample size, focus on a single geographical region, and lack of functional validation should be considered when interpreting these findings. Future studies in larger and more diverse populations, incorporating established PrC susceptibility loci, additional molecular biomarkers, and clinical risk factors, will be necessary to improve predictive performance and assess clinical utility. Nevertheless, the present findings support the value of pathway-based polygenic approaches and provide a foundation for further investigation of miRNA biogenesis genes in PrC risk stratification.

## Figures and Tables

**Table 1:** Characteristics of the study population.

**Characteristic**	**PrC Cases**	Controls
Total	532	505
Age, years, mean ± SD	66.74 ± 8.13	66.38 ± 5.19
TNM stage		
I–II, n (%)	296 (55.7)	–
III–IV, n (%)	236 (44.3)	–
PSA, mean (range)	15.38 (0.34–63.15)	2.06 (0.01–3.99)
Gleason Score, n (%)		
<8	317 (59.6)	–
≥8	215 (40.4)	–

Note: TNM—stage according to the Tumor–Node–Metastasis classification system; numbers in parentheses indicate percentages.

**Table 2:** Association analysis of variants in miRNA biogenesis pathway genes with prostate cancer susceptibility under the age- and ethnicity-adjusted model.

Chr	Position	Gene	SNP	EA	MAF		P_HWE_	OR (95% CI)	P	P_FDR_
					Controls	Cases				
1	35914532	*AGO1*	rs595055	C	0.32	0.23	0.078	0.64 (0.52–0.79)	2.49 × 10^−5^	6.72 × 10^−4^
1	111753752	*INKA2*	rs11584657	A	0.19	0.22	0.004	1.26 (1–1.58)	0.052	0.279
1	111766331	*DDX20*	rs197412	C	0.48	0.48	1.000	1 (0.83–1.19)	0.983	0.983
5	31400896	*DROSHA*	rs642321	A	0.28	0.23	0.200	0.87 (0.71–1.07)	0.182	0.400
5	31401340	*DROSHA*	rs10719	T	0.32	0.27	0.095	0.88 (0.72–1.06)	0.177	0.400
5	31435520	*DROSHA*	rs4867329	G	0.47	0.49	0.666	1.09 (0.92–1.3)	0.312	0.492
5	31532682	*DROSHA*	rs17409893	G	0.30	0.31	0.761	1.04 (0.86–1.27)	0.654	0.838
6	43524840	*XPO5*	rs2257082	T	0.35	0.35	1.000	1 (0.83–1.21)	0.963	0.983
8	140545763	*AGO2*	rs3864659	G	0.14	0.14	1.000	1.05 (0.81–1.35)	0.714	0.839
8	140584361	*AGO2*	rs7005286	A	0.22	0.19	0.253	0.89 (0.72–1.11)	0.295	0.492
12	53991815	*MIR196A2*	rs11614913	T	0.39	0.37	0.529	0.88 (0.74–1.06)	0.177	0.400
12	130367629	*PIWIL1*	rs11060845	T	0.09	0.05	0.010	0.65 (0.45–0.95)	0.024	0.164
12	130371771	*PIWIL1*	rs10773771	C	0.43	0.44	0.335	1.04 (0.87–1.25)	0.665	0.838
12	130871001	*RAN*	rs3809142	T	0.11	0.11	0.072	0.94 (0.7–1.26)	0.683	0.838
14	95090410	*DICER1*	rs13078	A	0.16	0.20	0.755	1.12 (0.89–1.41)	0.328	0.492
17	740186	*TLCD3A*	rs2740351	G	0.37	0.41	0.035	1.14 (0.95–1.35)	0.159	0.400
17	744946	*GEMIN4*	rs7813	C	0.37	0.42	0.228	1.24 (1.03–1.48)	0.021	0.164
17	745992	*GEMIN4*	rs3744741	A	0.18	0.15	0.570	0.83 (0.64–1.06)	0.133	0.400
17	746265	*GEMIN4*	rs4968104	A	0.21	0.22	0.432	1.06 (0.85–1.32)	0.588	0.836
17	746695	*GEMIN4*	rs2740348	G	0.16	0.20	0.141	1.34 (1.07–1.7)	0.012	0.164
17	30117165	*MIR423*	rs6505162	A	0.45	0.46	0.015	1.01 (0.84–1.2)	0.949	0.983
17	64506317	*DDX5*	rs1991401	C	0.38	0.41	0.029	1.14 (0.96–1.36)	0.134	0.400
19	13836478	*MIR27A*	rs895819	G	0.36	0.33	0.025	0.91 (0.76–1.09)	0.290	0.492
22	20110836	*DGCR8*	rs1640299	G	0.38	0.41	0.585	1.15 (0.96–1.38)	0.140	0.400
22	20111021	*DGCR8*	rs417309	A	0.09	0.10	0.210	1.19 (0.87–1.61)	0.273	0.492
22	20111059	*DGCR8*	rs720012	A	0.20	0.20	0.220	1 (0.8–1.27)	0.975	0.983
22	20111359	*DGCR8*	rs720014	C	0.21	0.23	0.039	1.16 (0.93–1.46)	0.193	0.400

Note: Chr—chromosome, Position—genomic position according to Genome Reference Consortium Human Build 38, SNP—single nucleotide polymorphism, EA—effect allele, MAF—minor allele frequency, P_HWE_—significance level for Hardy–Weinberg equilibrium test, OR—odds ratio, 95% CI—95% confidence interval, P—nominal significance level, P_FDR_—Benjamini–Hochberg false discovery rate-adjusted significance level.

**Table 3:** Performance of weighted and unweighted polygenic risk score models across ten repeated stratified training-validation splits.

Dataset	Model	Median AUC	IQR
Training	Weighted PRS	0.628	0.625–0.636
	Age + ethnicity	0.661	0.661–0.665
	Age + ethnicity + weighted PRS	0.704	0.703–0.709
Validation	Weighted PRS	0.553	0.542–0.578
	Age + ethnicity	0.660	0.654–0.663
	Age + ethnicity + weighted PRS	0.669	0.667–0.675
Training	Age + ethnicity + unweighted PRS	0.697	0.696–0.699
	Unweighted PRS	0.614	0.607–0.620
Validation	Age + ethnicity + unweighted PRS	0.670	0.667–0.674
	Unweighted PRS	0.547	0.524–0.564

Note: AUC—area under the curve; IQR—interquartile range; PRS—polygenic risk score.

**Table 4:** Statistical power for a case–control study of prostate cancer miRNA machinery gene variants.

Risk Allele Frequency	Power				
	OR = 1.1	OR = 1.2	OR = 1.3	OR = 1.4	OR = 1.5
0.1	11%	28%	53%	75%	90%
0.2	16%	45%	76%	93%	99%
0.3	20%	54%	85%	97%	99%
0.4	21%	60%	89%	98%	99%
0.5	21%	60%	89%	98%	99%

## Data Availability

The data that support the findings of this study are available from the corresponding author upon reasonable request.

## Supplementary Materials

The following supporting information can be downloaded at: https://www.mdpi.com/article/10.3390/ijms27125578/s1.

## Abbreviations

The following abbreviations are used in this manuscript:

PrC	Prostate cancer
PRS	Polygenic score
SNP	Single nucleotide polymorphism
miRNA	microRNA
GWAS	Genome-wide association study
QC	Quality control
CI	Confidence interval
LD	Linkage disequilibrium
ROC	Receiver operating characteristic
AUC	Area under the curve
OR	Odds ratio
FDR	False discovery rate
PSA	Prostate-specific antigen

## References

[1] Ferlay J., Colombet M., Soerjomataram I., Parkin D.M., Piñeros M., Znaor A., Bray F. (2021). Cancer statistics for the year 2020: An overview. Int. J. Cancer.

[2] Kaprin A.D., Starinsky V.V., Shakhzadova A.O. (2025). The State of Oncological Care for the Population of Russia in 2024.

[3] Hu Y.L., Zhang Y.J., Lv X.Y., Liu R.L., Zhong Z.H., Fu L.J., Bao M.H., Geng L.H., Xu H.J., Yu S.M. (2024). Impact of Omicron Variant Infection on Female Fertility and Laboratory Outcomes: A Self-Controlled Study. Am. J. Reprod. Immunol..

[4] Króliczewski J., Sobolewska A., Lejnowski D., Collawn J.F., Bartoszewski R. (2018). microRNA single polynucleotide polymorphism influences on microRNA biogenesis and mRNA target specificity. Gene.

[5] Luo X., Wen W. (2024). MicroRNA in prostate cancer: From biogenesis to applicative potential. BMC Urol..

[6] Wen J., Lv Z., Ding H., Fang X., Sun M. (2018). Association of miRNA biosynthesis genes DROSHA and DGCR8 polymorphisms with cancer susceptibility: A systematic review and meta-analysis. Biosci. Rep..

[7] Shao Y., Shen Y., Zhao L., Guo X., Niu C., Liu F. (2020). Association of microRNA biosynthesis genes XPO5 and RAN polymorphisms with cancer susceptibility: Bayesian hierarchical meta-analysis. J. Cancer.

[8] Yuan L., Chu H., Wang M., Gu X., Shi D., Ma L., Zhong D., Du M., Li P., Tong N. (2013). Genetic variation in DROSHA 3’UTR regulated by hsa-miR-27b is associated with bladder cancer risk. PLoS ONE.

[9] Bian X.J., Zhang G.M., Gu C.Y., Cai Y., Wang C.F., Shen Y.J., Zhu Y., Zhang H.L., Dai B., Ye D.W. (2014). Down-regulation of Dicer and Ago2 is associated with cell proliferation and apoptosis in prostate cancer. Tumour Biol..

[10] Nikolić Z., Savić Pavićević D., Vučić N., Cerović S., Vukotić V., Brajušković G. (2017). Genetic variants in RNA-induced silencing complex genes and prostate cancer. World J. Urol..

[11] Liu J., Liu J., Wei M., He Y., Liao B., Liao G., Li H., Huang J. (2012). Genetic variants in the microRNA machinery gene GEMIN4 are associated with risk of prostate cancer: A case-control study of the Chinese Han population. DNA Cell Biol..

[12] Hashemi M., Moradi N., Ziaee S.A., Narouie B., Soltani M.H., Rezaei M., Shahkar G., Taheri M. (2016). Association between single nucleotide polymorphism in miR-499, miR-196a2, miR-146a and miR-149 and prostate cancer risk in a sample of Iranian population. J. Adv. Res..

[13] Khusnutdinova E.K., Bermisheva M.A., Kutuev I.A., Yunusbayev B.B., Villems R., Dobretsov N., Kolchanov N., Rozanov A., Zavarzin G. (2008). Genetic Landscape of the Central Asia and Volga–Ural Region. Biosphere Origin and Evolution.

[14] Kang J., Brajanovski N., Chan K.T., Xuan J., Pearson R.B., Sanij E. (2021). Ribosomal proteins and human diseases: Molecular mechanisms and targeted therapy. Signal Transduct. Target. Ther..

[15] Li N., Spetz M.R., Ho M. (2020). The Role of Glypicans in Cancer Progression and Therapy. J. Histochem. Cytochem..

[16] Akinmuleya O.I., Cohen P.F., Kairemo K. (2024). 68Ga-PSMA PET CT/MRI in the initial diagnosis and staging of prostate cancer: A review. Adv. Radiother. Nucl. Med..

[17] Baker E., Schmidt K.M., Sims R., O’Donovan M.C., Williams J., Holmans P., Escott-Price V., GERAD Consortium (2018). POLARIS: Polygenic LD-adjusted risk score approach for set-based analysis of GWAS data. Genet. Epidemiol..

[18] de la Calle C.M., Bhanji Y., Pavlovich C.P., Isaacs W.B. (2022). The role of genetic testing in prostate cancer screening, diagnosis, and treatment. Curr. Opin. Oncol..

[19] Schumacher F.R., Al Olama A.A., Berndt S.I., Benlloch S., Ahmed M., Saunders E.J., Dadaev T., Leongamornlert D., Anokian E., Cieza-Borrella C. (2018). Association analyses of more than 140,000 men identify 63 new prostate cancer susceptibility loci. Nat. Genet..

[20] Castro E., Eeles R. (2012). The role of BRCA1 and BRCA2 in prostate cancer. Asian J. Androl..

[21] Nyberg T., Frost D., Barrowdale D., Evans D.G., Bancroft E., Adlard J., Ahmed M., Barwell J., Brady A.F., Brewer C. (2020). Prostate Cancer Risks for Male BRCA1 and BRCA2 Mutation Carriers: A Prospective Cohort Study. Eur. Urol..

[22] Barnes D.R., Silvestri V., Leslie G., McGuffog L., Dennis J., Yang X., Adlard J., Agnarsson B.A., Ahmed M., Aittomäki K. (2022). Breast and Prostate Cancer Risks for Male BRCA1 and BRCA2 Pathogenic Variant Carriers Using Polygenic Risk Scores. J. Natl. Cancer Inst..

[23] Klein R.J., Vertosick E., Sjoberg D., Ulmert D., Rönn A.C., Häggström C., Thysell E., Hallmans G., Dahlin A., Stattin P. (2022). Prostate cancer polygenic risk score and prediction of lethal prostate cancer. npj Precis. Oncol..

[24] Chen F., Madduri R.K., Rodriguez A.A., Darst B.F., Chou A., Sheng X., Wang A., Shen J., Saunders E.J., Rhie S.K. (2023). Evidence of Novel Susceptibility Variants for Prostate Cancer and a Multiancestry Polygenic Risk Score Associated with Aggressive Disease in Men of African Ancestry. Eur. Urol..

[25] Zhou Z., Zhuo L., Fu X., Lv J., Zou Q., Qi R. (2024). Joint masking and self-supervised strategies for inferring small molecule-miRNA associations. Mol. Ther. Nucleic Acids.

[26] Yang Z., Ping Y.Q., Wang M.W., Zhang C., Zhou S.H., Xi Y.T., Zhu K.K., Ding W., Zhang Q.Y., Song Z.C. (2025). Identification, structure, and agonist design of an androgen membrane receptor. Cell.

[27] Li D., Li F., Meng L., Wei H., Zhang Q., Jiang F., Chen D.N., Li W., Tan Y.Q., Li J.D. (2021). RNF216 regulates meiosis and PKA stability in the testes. FASEB J..

[28] Espinoza-Sánchez N.A., Götte M. (2020). Role of cell surface proteoglycans in cancer immunotherapy. Semin. Cancer Biol..

[29] World Medical Association (2013). World Medical Association Declaration of Helsinki: Ethical principles for medical research involving human subjects. Jama.

[30] Ivanova E., Gilyazova I., Pavlov V., Izmailov A., Gimalova G., Karunas A., Prokopenko I., Khusnutdinova E. (2022). MicroRNA Processing Pathway-Based Polygenic Score for Clear Cell Renal Cell Carcinoma in the Volga-Ural Region Populations of Eurasian Continent. Genes.

[31] Sherry S.T., Ward M., Sirotkin K. (1999). dbSNP-database for single nucleotide polymorphisms and other classes of minor genetic variation. Genome Res..

[32] Gibbs R.A., Belmont J.W., Hardenbol P., Willis T.D., Yu F.L., Yang H.M., Ch'ang L.Y., Huang W., Liu B., Shen Y. (2003). The International HapMap Project. Nature.

[33] Griffiths-Jones S., Grocock R.J., van Dongen S., Bateman A., Enright A.J. (2006). miRBase: microRNA sequences, targets and gene nomenclature. Nucleic Acids Res..

[34] Howe K.L., Achuthan P., Allen J., Allen J., Alvarez-Jarreta J., Amode M.R., Armean I.M., Azov A.G., Bennett R., Bhai J. (2021). Ensembl 2021. Nucleic Acids Res..

[35] Purcell S., Neale B., Todd-Brown K., Thomas L., Ferreira M.A., Bender D., Maller J., Sklar P., de Bakker P.I., Daly M.J. (2007). PLINK: A tool set for whole-genome association and population-based linkage analyses. Am. J. Hum. Genet..

[36] Purcell S., Cherny S.S., Sham P.C. (2003). Genetic Power Calculator: Design of linkage and association genetic mapping studies of complex traits. Bioinformatics.

[37] Gauderman W.J. (2002). Sample size requirements for matched case-control studies of gene-environment interaction. Stat. Med..

[38] Benjamini Y., Hochberg Y. (1995). Controlling the False Discovery Rate—A Practical and Powerful Approach to Multiple Testing. J. R. Stat. Soc. Ser. B.

[39] Robin X., Turck N., Hainard A., Tiberti N., Lisacek F., Sanchez J.C., Muller M. (2011). pROC: An open-source package for R and S+ to analyze and compare ROC curves. BMC Bioinform..

